# Type 3 Diabetes: Cross Talk between Differentially Regulated Proteins of Type 2 Diabetes Mellitus and Alzheimer’s Disease

**DOI:** 10.1038/srep25589

**Published:** 2016-05-06

**Authors:** Khyati Mittal, Ruchi Jakhmola Mani, Deepshikha Pande Katare

**Affiliations:** 1 Proteomic & Translational Research Lab, Centre for Medical Biotechnology, Amity Institute of Biotechnology, Amity University Uttar Pradesh, Noida 201313, India

## Abstract

Type 3 Diabetes (T3D) is a neuroendocrine disorder that represents the progression of Type 2 Diabetes Mellitus (T2DM) to Alzheimer’s disease (AD). T3D contributes in the increase of the total load of Alzheimer’s patients worldwide. The protein network based strategies were used for the analysis of protein interactions and hypothesis was derived describing the possible routes of communications among proteins. The hypothesis provides the insight on the probable mechanism of the disease progression for T3D. The current study also suggests that insulin degrading enzyme (IDE) could be the major player which holds the capacity to shift T2DM to T3D by altering metabolic pathways like regulation of beta-cell development, negative regulation of PI3K/AKT pathways and amyloid beta degradation.

Insulin signaling pathways are conserved in various types of cells and tissues. It regulates the energy metabolism, homeostasis and reproduction in living system. It reaches the brain via cerebral spinal fluid and transporters present at the blood brain barrier. It is proposed to enhance cognitive abilities via activation of insulin receptors in the hippocampal region of brain. It stimulates translocation of GLUT4 to hippocampal plasma membranes thereby enhancing the glucose uptake in the time dependent manner^1^. Glucose utilization during neuronal activity is similar in both peripheral tissue and hippocampal region^1^. Scientists have worked extensively to understand the molecular mechanisms involved in the production and secretion of insulin in the brain and pancreas^2^. Their findings suggest that both beta cells and neurons respond to glucose and hormonal stimuli by depolarization of ATP sensitive potassium channels in similar fashion. Few studies report that insulin was stored in synaptic vesicles at nerve endings in rat brain and was released under depolarization conditions^2^. The study also suggests that insulin secretion in synaptosomes is increased by glucose and addition of glycolytic inhibitor resulted in 50% decrease in glucose-induced release of immunoreactive insulin^2^. Hence the process of glucose metabolism is similar in brain and pancreas and the brain itself might synthesize some portion of the insulin^2^.

The binding of insulin to its receptor leads to cascades of intracellular signaling which activates the Insulin Receptor Substrate-1(IRS1), extracellular signal-related kinase/mitogen -activated protein kinase (ERK/MAPK), and PI3kinase/AKT pathways (PI3K/AKT) followed by inhibition or suppression of glycogen synthase kinase-3 (GSK-3)^2^. Disturbances to these pathways can lead to complication like cardiovascular diseases, pancreatic cancer, neuropathy, nephropathy etc^2^. It also adds to several other issues like mitochondrial dysfunction, oxidative stress and dysregulated metabolic profiles^2^.

There is an exponential increase in the prevalence of T2DM cases worldwide and it is likely to reach 592 million by 2035^3^. Also the incidences of T2DM induced AD is rapidly increasing in human population in last few years^4^. T2DM patients have almost double the chances of developing AD in comparison to the patients that have only insulin resistance^5^. Therefore, T3D is also adding to the already existing burden of AD in the society.

T2DM and AD patients have similar amyloid beta deposits both in pancreas as in the brain^6^. Several researchers have suggested this new pathology to be addressed as Type 3 Diabetes (T3D)^4^,^5^,^6^,^7^. Some of the target receptors of T2DM such as IGF-1R, PPARG and IDE are also involved in the regulation of the expression and phosphorylation of tau protein^7^. It is intriguing to observe that both hyperinsulinaemia and IDE are related to the risk of AD and is independent of APOE4 gene^7^. Hence T2DM induced AD is believed to be sporadic irrespective of presence of heterozygous or homozygous conditions of ApoE^2^,^7^.

Seventy susceptible genes are associated with T2DM at a genome-wide level^8^. Some of the polymorphic genes associated with T2DM are PPARG, KCNJ11, TCF7L2, HHEX/IDE, CDKAL1, SLC30A8, IRS1, INSR etc^8^.

In the current study, we report for the first time an ideal hypothesis relating possible protein-protein interactions that might be taken up by the system during the progression of T2DM induced AD. It also predicts candidate/s for developing drugs that can target both pathological conditions.

## Results

**Figure 1:**
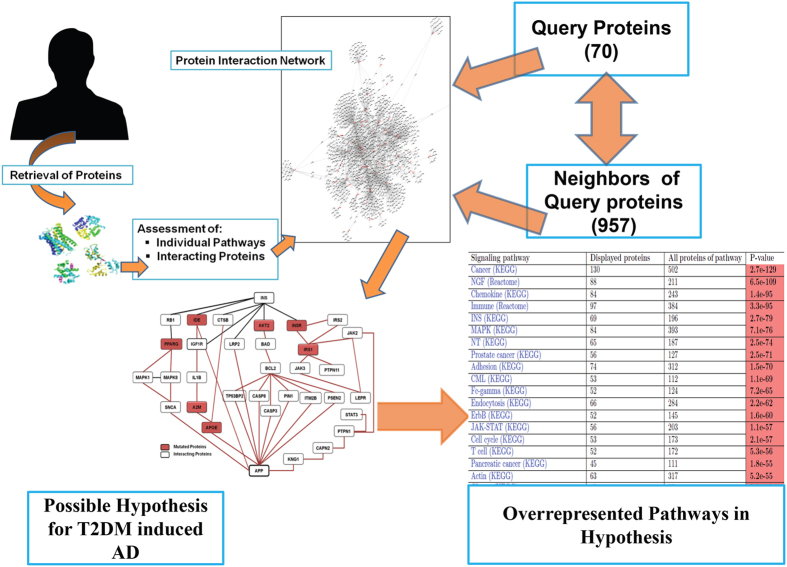
Interactions between differentially expressed proteins of AD and T2DM. The figure shows the methodology of work done. It correlates the differentially expressed proteins of Type 2 diabetes and Alzheimer’s disease. The table represents the majorly involved pathways in T3D pathology and ranked according to their predicted P-values.

Few differentially regulated proteins of T2DM and AD were collected after extensive literature mining (Supplementary Table 1). The proteins were queried on Pathwaylinker2.0 and the interactions were displayed as balls and sticks. Balls are the queried proteins and sticks represent the interactions between them, both theoretical and experimental (Fig. 1).

These interactions indicate the cross talk between proteins. This is either due to their participation in similar pathways or because of their intermittent and short lived interactions in some processes. The connections were also established on the basis of the shared protein partners.

A total of 957 interacting proteins were retrieved for 70 query proteins. Entire subset of 1027 proteins was analyzed for overrepresented and shared pathways. The biasedness of the data was calculated statistically by examining P-values. For example 196 proteins are involved in Insulin (INS) Pathway (HPRD, STRING, BioGrid) (Table 1) and in the dataset of 1027 proteins only 69 proteins are involved in INS Pathway. The probability of coming INS pathway in our dataset is 2.7e79 which means INS pathway is among the most followed pathway by both query and neighbor proteins. Similarly the probability of occurrence of Alzheimer’s and Diabetes II pathways in our dataset is 6.6e-41 and 6.9e-35 respectively.

It was seen that Chemokine (AKT1 [P31749]; AKT2 [P31751]; CCL4 [P13236]), Immune (CD4 [P01730]; VCAM1 [P19320]; PTPN11 [Q06124]), INS (INSR [P06213]; INS [P01308]; IRS1 [P35568]) MAPK (MAPK1 [P28482]; MAPK8 [P45983]; EGFR [P00533]), Cell cycle (YWHAZ [P63104]; RB1 [P06400]; GSK3B [P49841]), JAK-STAT (JAK2 [O60674] ; JAK3 [P52333] ; STAT3 [P40763]), Pancreatic Cancer (BAD [Q92934]; MAPK8 [P45983]; STAT3 [P40763]), T-Cell (GSK3B [P49841]); PIK3R3 [Q92569]; JUN [P05412]), Fc-epsilon (IL4 [P05112]; RAF1 [P04049]; GRB2 [P62993]), P53 (IGF1 [P05019]; CDK4 [P11802]; BAX [Q07812]), Alzheimer (APP [P05067]; IDE [P14735]; BACE2 [Q9Y5Z0]), Apoptosis (BCL2 [P10415]; IL1B [P01584]; CASP8 [Q14790]) and SCLC (BCL2 [P10415]; E2F1 [Q01094]; CDK6 [Q00534]) were some of the overrepresented signaling pathways with their P-values (Table 1). Hence the sub-pathways and the other processes under these Super-Pathways are predicted to take place in the Type 3 pathology (Supplementary Table 2).

**Figure 2:**
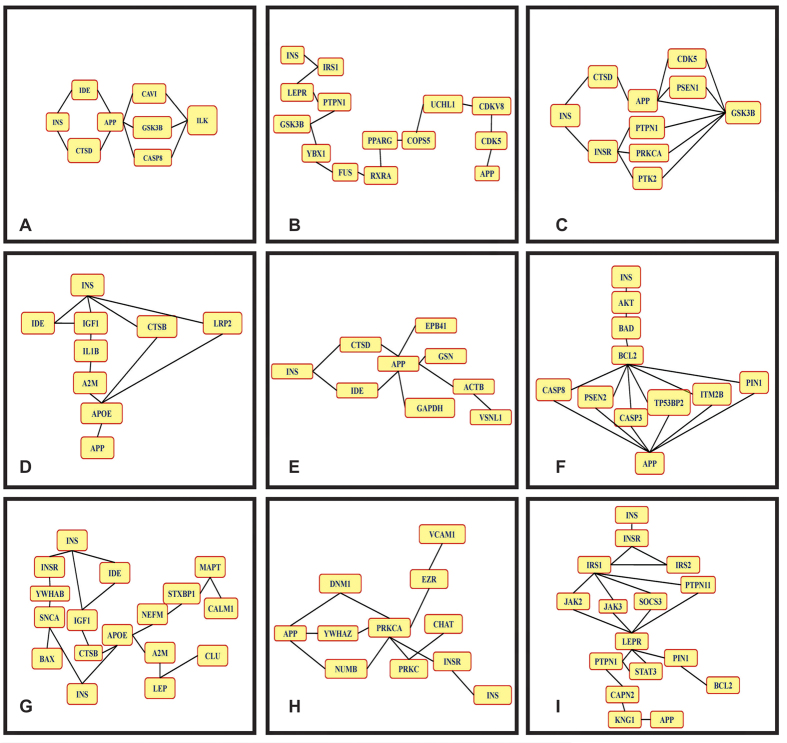
Schematic Representation of different protein interactions involved in T2DM induced AD. The figure shows the different hypothesis of progression of T3D. These short interactions depict the mechanism through which insulin and amyloid beta are linked.

Table 1 represents A (Query proteins) and B (Proteins involved in the specific pathways as per databases like STRING, HPRD and BIOGRID). Several hypotheses were framed by using the interacting proteins data. These small interactions depict the probable routes through which insulin resistance might lead to amyloid plaque formation in brain. These interactions indirectly or directly affect each other’s function and contribute in disease pathology (Fig. 2A–I).

**Figure 3:**
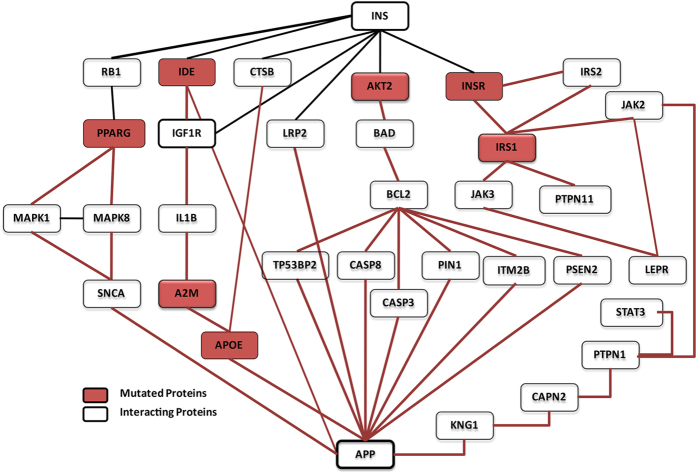
Interaction of selected proteins from the network supposedly followed in T3D (mutated proteins are highlighted in red). Final protein- interaction network was framed which includes mutated and differentially expressed proteins which link Type 2 Diabetes and Alzheimer’s disease.

**Figure 4:**
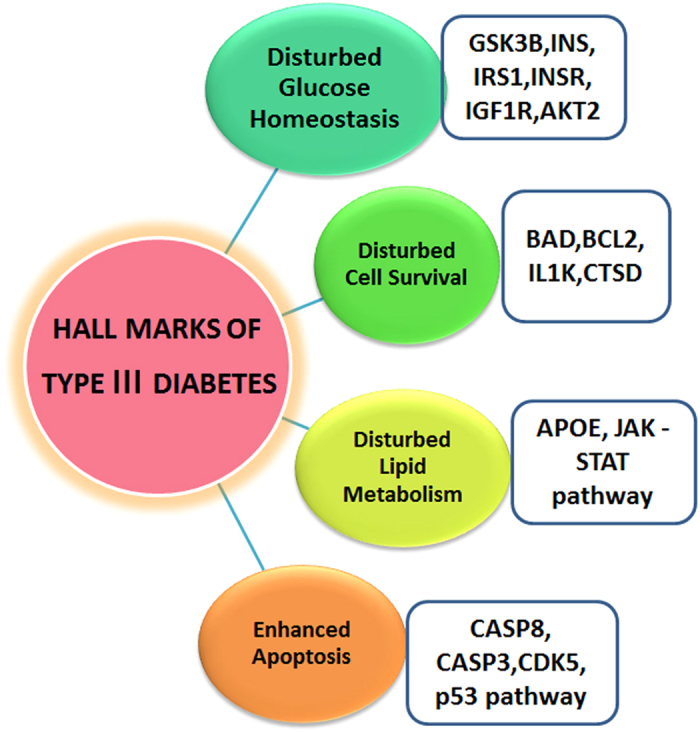
Hallmarks of Type 3 Diabetes. Attributes of Type 3 Diabetes represents the disturbed metabolic processes and pathways in Type 3 diabetes.

For the identification of potential druggable targets a common hypothesis was designed for both nearest and distant interacting partners. This hypothesis contains seven possible routes of progression of T2DM to AD. The role/function of proteins in hypothesis (Figs 3 and 4) is discussed below:

### Route I

Diabetes is the result of impaired insulin signal transduction which up regulates the activity of IDE^9^,^10^. IDE degrade both insulin and amylin in a dose dependent manner^11^. It is proposed here that since IDE is elevated in both T2DM and AD, its increased levels degrade insulin^10^. The elevated expression of IDE downregulates insulin growth factor-1 (IGF-1) which further increases the activity of interleukin 1 beta (IL1B)^12^,^13^. Excess of interleukins produces oxidative stress in the brain. Various studies report that IL1B forms a complex with alpha- 2-microglobulin (A2M)^14^,^15^ which controls the activity of apolipoprotein E (APOE) involved in the formation of amyloid precursor protein (APP) and hence contributes in the pathology of Alzheimer’s disease^16^.

### Route II

It is proposed that the insulin resistance causes disturbances in insulin signaling pathway which retains RAC-beta serine/threonine-protein kinase (AKT)^17^ in dephosphorylated state followed by de-phosphorylation of Bcl2-associated agonist of cell death (BAD)^18^. Dephosphorylation causes BAD to initiate apoptosis process^19^. The BAD protein plays key role in both diabetes and AD pathology. In T2DM the dephosphorylated BAD upregulates BCl2 and causes cell death. Similarly in AD, dephosphorylated BAD protein causes mitochondrial dysfunction^20^ thereby inducing apoptosis through Apoptosis regulator Bcl-2 (BCL2) mediated proteins such as CASP3^21^,^22^, CASP8^23^,^24^, PSEN1^25^,^26^, PIN1^27^,^28^, TP53BP2^29^,^30^, ITM2B^31^,^32^ and results in APP formation that contributes in the pathogenesis of Alzheimer’s disease in the early stage.

### Route III

Cathepsin B (CTSB) is involved in the conversion of proinsulin to insulin^33^. It is proposed that the insulin resistance causes down regulation of CTSB protein and hence disturbs the insulin balance in the system. The CTSB protein further interacts with APOE and up regulates it^34^. Increased APOE expression results in APP formation and hence also contributes in AD pathology.

### Route IV

Low density lipoprotein 2(LRP2) proteins helps in the retention of insulin from kidney during clearance of other substances (termed as clearance pathway)^35^. It is proposed that the decreased insulin signal causes decreased expression of LRP2 and hence eventually up regulates the expression of APOE thereby participating in AD pathology^36^.

### Route V

Retinoblastoma protein (RB) has been shown to facilitate adipocyte differentiation by forming a complex with insulin^37^,^38^. It is proposed that the Insulin resistance inhibits this complex formation, which in turn affects the RB protein. This RB protein further prevents PPARG from inhibition^39^. PPARG activation is linked with MAPK signaling pathway, up regulation of former results in decrease activity of Mitogen activated protein kinases1 (MAPK1) and Mitogen activated protein kinase 8 (MAPK8)^40^. The decreased activity of MAPK pathway affects the alpha synuclein (SNCA)^41^ and this SNCA interacts with amyloid beta and contributes in AD^42^.

### Route VI

It is proposed that the impairment in the insulin signaling reduces the affinity of insulin for Insulin receptor (INSR)^43^. INSR interacts with Insulin receptor substrate1(IRS1) and Insulin receptor substrate2 (IRS2), although the nature of interaction between the two has not been understood yet^44^,^45^. Changes in activity of IRS2 affect IRS1^46^. Further IRS1 affects regulatory receptor for fat metabolism i.e. leptin receptor (LEPR) through JAK3^47^,^48^, SOCS3^49^,^50^, JAK2^51^,^52^ and PTPN11^53^,^54^ and LEPR protein interacts with PIN1^55^. PIN1 in turn regulates the amyloid-β production^28^.

### Route VII

It is proposed that the interaction of PTPN1 with LEPR is indirect through JAK2^56^,^34^ and STAT3^57^,^34^. In some studies PTPN1 is also involved in formation of APP via CAPN2^58^ followed by KNG1^59^,^60^ signaling. It was also seen that impaired signals of IRS1 are directly involved in the progression of AD via BCL-2 protein.

From the above mentioned hypotheses, the closest and most appropriate route for progression of T2DM to AD is Route I. This route was chosen because it has those proteins which are directly involved in pathogenesis of both T2DM and AD respectively. Insulin resistance ultimately leads to destruction of beta-cells of pancreas^4^. Therefore in the later stages of diabetes mellitus, the levels of insulin in the body start depleting^4^. Decreased insulin concentrations cause defects in cognition, memory and learning abilities of an individual similar to Alzheimer’s patients. On the other hand, in diseased condition the overproduced amylin reaches the brain via blood brain barrier and form plaques similar to amyloid beta. The deposition of amylin in brain blood vessels is responsible for the building of other amylin amyloid plaques and hence increases the risk of AD. These deposits were also seen in the brain tissue of older people who had diabetes and vascular dementia. In hyperinsulinemic condition, the variation of IDE results in the degradation of insulin rather than amyloid beta^10^,^13^. Therefore two events make this pathology worse, first is excessive degradation of insulin due to overexpression of IDE in T2DM which in turn accounts for lesser levels of insulin in brain and secondly, the inefficient clearance of ABeta in the brain by IDE^10^,^13^.

## Discussion

The study deciphered the series of interactions which may possibly happen during the pathology of T3D. Out of the seven possible routes of the progression of disease the most appropriate is the one which initiates with the signal transduction through IDE receptor. As mentioned in route 1 the transmission of impaired insulin signal upregulates IDE and increases its activity^12^. Alternatively there are studies which report that IDE is mutated in T2DM^10^. It is very likely that any mutations in IDE changes its function and disturbs various signaling processes (Supplementary table 2) and these disturbances can contribute to T3D pathology.

Increased IDE activity causes more degradation of insulin than the normal conditions^13^. Some studies reported that glucose utilization by brain is independent of insulin but on the contrary Ren *et al.*^1^ have confirmed that GLUT 4 constitutes a subset of neurons in hippocampal region which are insulin sensitive. Uptake of the glucose through these receptors might play an important role in the cognition and hippocampal based learning^1^. IDE inhibitors can be a good therapeutic intervention to stop these chains of events at this level^11^. Uncontrolled IDE activity further downregulates IGF-1R protein expression^12^. IGF-1R is important for synaptic transfer and plasticity. Its downregulation will lead to neuronal damage. According to the study of Westwood *et al.*^9^, IGF-1R downregulation in brain causes formation of oligomers and cognitive impairment which increases the chances of developing Alzheimer’s disease in a diabetic patient. Higher expression of IGF-1R also contributes in development of AD in older people. Therefore both the upregulation and downregulation of IGF-1R affects brain. Though IGF-1R is a druggable target, therapies based on the growth factor failed in clinical trials. Improvisations in these drugs are still awaited. IGF-1R may further activate interleukins and creates excessive oxidative stress in the brain tissue^13^. Oxidative stress is already implicated as one of the factors for deposition of amyloid beta in brain.

The study of Maedler *et al.*^15^ reported that disturbed IL1B signals are involved in the progression of insulin resistance and leads to T2DM. These activated interleukins and alpha-2-microglobuin then forms a complex and eventually upregulates the expression of APOE^16^. Therapies like anti-APOE antibody are already under clinical trials for decreasing amyloid beta load in the AD brain.

Drug discovery groups have a wide-spread understanding now that the incurable diseases have multiple molecular targets. Hence drugs directed to single molecular target (conventional old drugs) are insufficient. To accomplish this objective one can either target multiple molecular targets at the same time using different combinations of drug or use one drug which controls multiple targets. Both the approaches are equally achievable from the clinical perspective.

In the past many multi target drugs have been proposed for both T2DM and AD respectively. Till date there are no drugs available for Type 3 Diabetes as no receptor has been worked out so far. Therefore the present study concludes that proteins involved in Route 1 may provide some insight into the mechanism of the disease. Identified major proteins i.e. IDE, IGF-1R, IL1B, A2M and APOE can be targeted simultaneously or individually to target this disease. Since these events start with dysregulations of signals at IDE level, it is proposed that IDE should be the favored target and could be an answer to this incurable disease.

## Methods

### Development of a local protein database for Network Assessment

Extensive literature mining was done to retrieve the list of proteins which are differentially regulated in T2DM and AD. Accession numbers of all the proteins were retrieved from UniprotKB (http://www.uniprot.org/) and stored in a local database (Supplementary Table 1). These proteins were checked with Gene Ontology Databases for their involvement in different metabolic processes.

### Generation of Protein- protein interaction Network

Retrieved proteins were queried with different databases like HPRD, BioGrid and STRING which contains information of experimentally validated protein- protein interactions identified by different biochemical assays. This will generate the list of protein interactors to these query proteins. The total subset of proteins was analyzed by Pathwaylinker2.0 (http://pathwaylinker.org/). It creates a master network among the queried proteins and their neighbors. This network was checked for the pathways mostly followed by total subset of 70 query as well as their neighbor proteins. This results in the list of pathways along with their P-values (Probability value). P-values ensured that the results were independent of each other. The selection of query proteins of both T2DM and AD was done randomly without the prior knowledge about their shared processes and pathways.

### Framing and analysis of Hypothesis

The complex master network was segregated into nine short interactions to understand the relationship between the queries and their neighboring partners. These interactions were merged keeping insulin as the starting point of the hypothesis which culminates at APP. Interacting neighbor proteins were chosen from the master network and added to the hypothesis in step-wise manner. The final hypothesis was created and explains the events that the system undertakes to shift from insulin resistance to formation of amyloid beta plaques.

## Additional Information

**How to cite this article**: Mittal, K. *et al.* Type 3 Diabetes: Cross Talk between Differentially Regulated Proteins of Type 2 Diabetes Mellitus and Alzheimer's Disease. *Sci. Rep.* **6**, 25589; doi: 10.1038/srep25589 (2016).

## Figures and Tables

**Table 1:** List of Pathways followed by total queried proteins and their first neighbors.

Signaling Pathway	Displayed Proteins (A)	All proteins of Pathway (B)	P-value
Chemokine(KEGG)	84	243	1.4e-95
Immune (Reactome)	97	384	3.3e-95
INS (KEGG)	69	196	2.7e-79
MAPK(KEGG)	84	393	7.1e-76
Cell Cycle(KEGG)	53	173	2.1e-57
JAK-STAT(KEGG)	56	203	1.1e-57
PancreaticCancer(KEGG)	45	111	1.8e-55
T cell (KEGG)	52	172	5.3e-56
Cytokine (KEGG)	62	323	3.6e-53
Fc-epsilon (KEGG)	41	101	1.1e-50
Apoptosis (KEGG)	42	134	3.1e-46
P53 (KEGG)	35	93	4.8e-42
Alzheimer (KEGG)	46	227	6.6e-41
Diabetes II (KEGG)	29	78	6.9e-35
SCLC(KEGG)	38	128	6.2e-41
